# Evidence for a multidimensional account of cognitive and affective theory of mind: A state-trace analysis

**DOI:** 10.3758/s13421-023-01481-9

**Published:** 2023-11-28

**Authors:** Amy L. Jarvis, Hannah A. D. Keage, Stephanie Wong, Michael Weightman, Rachel G. Stephens

**Affiliations:** 1 Justice and Society Unit, University of South Australia, Adelaide, Australia; 2 College of Education, Psychology and Social Work, Flinders University, Adelaide, Australia; 3 Faculty of Health and Medical Sciences, University of Adelaide, Adelaide, Australia; 4 College of Medicine and Public Health, Flinders University, Adelaide, Australia; 5 School of Psychology, University of Adelaide, Adelaide, Australia

**Keywords:** Theory of mind, Social cognition, Affective, Emotion, State-trace analysis

## Abstract

Theory of mind (ToM) has been argued to be a multidimensional construct, with ToM inferences depending on distinct processes across affective and cognitive ToM tasks and across first-order cognitive and second-order cognitive ToM tasks. Behavioural evidence for a multidimensional account has primarily depended on dissociations identified via analysis of variance, a statistical approach insufficient for assessing dimensionality. Instead, state-trace analysis (STA) is a more appropriate statistical technique to uncover dimensionality. The current study first applied STA to two summary datasets that had previously identified key dissociations between cognitive and affective ToM; these reanalyses did not support a multidimensional account of ToM. Next, STA was applied to a more detailed dataset to reveal whether ToM is based on multiple processes in a sample of 115 older adults aged 60–85 years (*M* = 68.5, *SD* = 5.92, 61.7% female) with higher or lower emotion perception ability. Participants made ToM judgements about different social exchanges (e.g., sarcasm or lying). STA results supported a multidimensional account of ToM across first-order cognitive, second-order cognitive, and affective ToM subdomains. These results lay a more rigorous foundation for subsequent studies to further examine the dimensionality of ToM and to apply formal modelling, progressing the field’s understanding and measurement of the cognitive processes driving ToM judgements.

**Supplementary Information:**

The online version contains supplementary material available at 10.3758/s13421-023-01481-9.

## Introduction

A core component of social cognition is theory of mind (ToM), the capacity to make inferences about the mental state of others, a cognitive ability proposed by some to be a multidimensional construct consisting of different subdomains including cognitive and affective ToM (e.g., Duval et al., 2011; Li et al., 2013; Shamay-Tsoory et al., 2010; Wang & Su, 2013). The affective ToM subdomain relates to inferences about the affective state, emotion, or feeling of others (Duval et al., 2011). The cognitive ToM subdomain involves inferences about the beliefs, thoughts, or intentions of others and can be further categorized into first- and second-order mental representations (Duval et al., 2011). First-order representations require adopting the perspective of a single individual (i.e., Person A believes . . .), while second-order representations involve simultaneously adopting the perspectives of two individuals (i.e., Person A believes that Berson B believes . . . ; Duval et al., 2011). Although these ToM subdomains correspond to different kinds of tasks or inferences, a key question is whether they are driven by different underlying cognitive processes.

A multidimensional account of ToM is supported by evidence for cognitive and affective ToM having different (i) developmental trajectories (Sebastian et al., 2012), (ii) recruitment of brain regions (Shamay-Tsoory & Aharon-Peretz, 2007), (iii) and impairments in clinical populations (Bora et al., 2009; Lin et al., 2021; Shamay-Tsoory et al., 2010; Weightman et al., 2014). Therefore, these differences between cognitive and affective ToM suggest that each subdomain involves distinct underlying processes. Nonetheless, ToM continues to be defined by some researchers as a unidimensional construct (e.g., Benito-Ruiz et al., 2022). Although the dimensionality of other cognitive domains, such as memory, attention, executive functioning, and reasoning is well established (Lezak et al., 2012; Robinson & Irwin, 2019; Stephens et al., 2018), research exploring the dimensionality of ToM is scarcer. Given that ToM deficits are associated with functional outcomes in clinical populations, including people with schizophrenia (Javed & Charles, 2018), traumatic brain injury (Ubukata et al., 2014), autism spectrum disorder (Bishop-Fitzpatrick et al., 2017), stroke (Adams et al., 2020), and bipolar disorder (Vlad et al., 2018), it is important to understand the processes that drive ToM performance, which can inform the development of theoretically sound measures.

Key evidence for the distinct dimensions of cognitive and affective ToM includes the observation of differential impairments across different age groups (Bottiroli et al., 2016; Sebastian et al., 2012) and clinical populations (Bora et al., 2009; Lin et al., 2021; Shamay-Tsoory et al., 2010; Weightman et al., 2014). Regarding ageing research, older adults show deficits in cognitive but not affective ToM compared with younger adults (Bottiroli et al., 2016), while affective but not cognitive ToM impairments have been identified in adolescents compared with healthy adults (Sebastian et al., 2012). Within clinical populations, meta-analytic results have reported more significant cognitive ToM impairments compared with healthy controls in patients with schizophrenia (Bora et al., 2009), while affective ToM is more impaired in patients with traumatic brain injury (Lin et al., 2021), major depressive disorder (Weightman et al., 2014), and psychopathy (Shamay-Tsoory et al., 2010).

One possible key driver of the distinction between cognitive and affective ToM processes could be that emotion perception is more closely associated with affective than cognitive ToM. Emotion perception involves the ability to accurately read information about the emotional state of someone through verbal, bodily, and facial cues and is suggested to recruit partially distinct brain regions to cognitive and affective ToM (Bek et al., 2021; Mitchell & Phillips, 2015). Studies have shown that emotion perception performance is significantly correlated with affective ToM performance only (Bek et al., 2021; Henry et al., 2006). However, it is currently unclear whether this correlation is due to shared emotion perception functions or due to similarities in task demands (i.e., identifying complex affective states versus basic emotions from face or eye region images; Bek et al., 2021; Henry et al., 2006).

The distinction between first- versus second-order cognitive ToM dimensions has also been supported by the observation of differential impairments (Duval et al., 2011, 2012). Compared with healthy controls and first-order cognitive ToM performance, significant second-order cognitive impairments have been demonstrated in some clinical populations, including traumatic brain injury (Bibby & McDonald, 2005) and dementia (Duval et al., 2012). Similar selective impairments are observed when comparing older adults with young or middle-aged adults (Duval et al., 2011). However, some researchers suggest that first- versus second-order cognitive ToM tasks differ in complexity and difficulty (e.g., Bibby & McDonald, 2005). Therefore, differential performance may not reflect separate subdimensions of cognitive ToM but instead could reflect the operation of a single dimension of cognitive ToM across different levels of task complexity.

An important limitation to previous behavioural results supporting ToM being multidimensional is that such evidence primarily relies on identifying dissociations between groups or tasks via statistical tests such as analyses of variance, *t* tests, or correlations (e.g., Bottiroli et al., 2016; Shamay-Tsoory et al., 2010; Warnell & Redcay, 2019). In contrast, state-trace analysis (STA), which tests for any departures from a monotonic relationship between two dependent variables (e.g., performance on cognitive vs. affective ToM tasks), is better suited to test for multiple underlying latent variables (Stephens et al., 2019). Standard dissociation evidence—that is, observing that a factor (e.g., age) affects performance on one task but has no (or a limited) effect on another task—is consistent with the notion of multiple dimensions but is neither necessary nor sufficient for inferring multiple dimensions (Newell & Dunn, 2008; Newell et al., 2010). Crucially, many key dissociations used to support multiple cognitive processes have been shown to depend heavily on the strong assumption of a linear mapping between latent and dependent variables, with the dissociations “disappearing” under state-trace analysis (see Stephens et al., 2019).

**Fig. 1:**
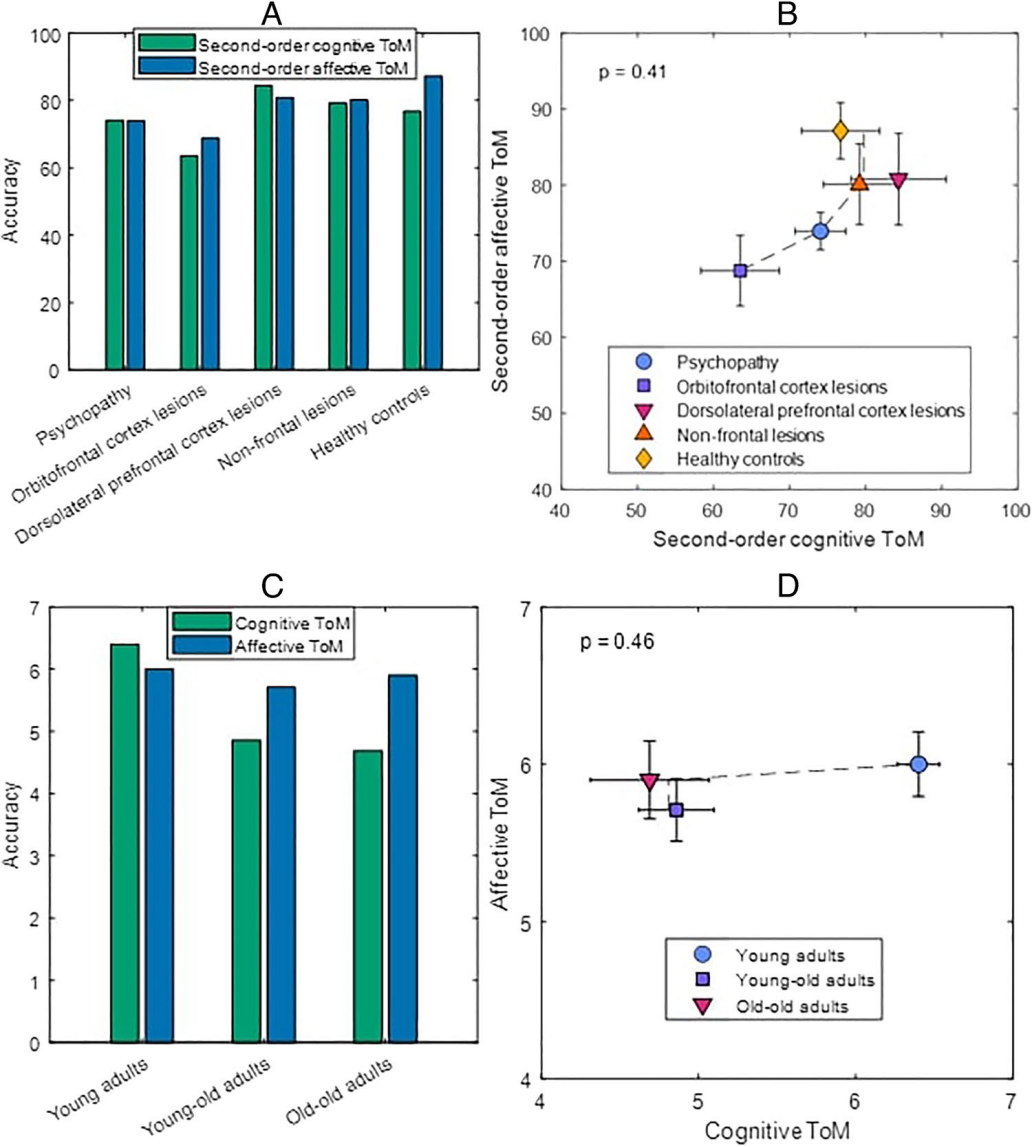
Key dissociation results (**A** and **C**) and corresponding state-trace plots (**B** and **D**) based on summary statistics (*M*, *SD*, *N*) extracted from previously published results (Bottiroli et al., 2016; Shamay-Tsoory et al., 2010). *Note.* **B** and **D**: error bars are *SE*s; the dashed line shows the best-fitting monotonic curve; the *p* values are based on conjoint monotonic regression, testing for significant departures from the best-fitting monotonic curve (*SD*s were used to estimate the variability of the data, assuming that observations were normally distributed). **A**–**B** present data from Shamay-Tsoory et al. (2010): mean accuracy for five groups of people on a second-order cognitive versus second-order affective ToM task. The researchers found an important dissociation: compared with healthy controls, the psychopathy group and those with orbitofrontal cortex (OFC) lesions were impaired for affective ToM but not for cognitive ToM (**A**; the OFC group was also impaired relative to the nonfrontal lesions group on affective ToM only). However, STA shows that the group means do not significantly depart from a monotonic curve, so they are consistent with a single latent variable (**B**). **C**–**D** present data from Bottiroli et al. (2016): Mean accuracy for three age groups on a cognitive versus affective ToM task. The researchers found a key dissociation: young adults scored more highly than both young–old and old–old adults on cognitive ToM but not affective ToM (**C**). However, the group means do not significantly depart from a monotonic curve, so they are consistent with a single latent variable (**D**). (Color figure online)

However, STA has not yet been applied to ToM data. STA involves examining a state-trace plot, in which participants’ mean responses on one dependent variable (e.g., responses on a cognitive ToM task) are plotted against their corresponding mean responses on another dependent variable (e.g., responses on an affective ToM task) across different experimental conditions or groups (Bamber, 1979; Dunn, 2008; Newell & Dunn, 2008; Stephens et al., 2019); see Fig. 1B and D for examples. The key idea is that if the two dependent variables are driven by a single latent psychological variable (e.g., one core ToM process), assuming only a monotonic mapping between this latent variable and the dependent variables, the two dependent variables must themselves be monotonically related. Therefore, if all data points on the state-trace plot do not fall on a single, monotonically increasing (or decreasing) curve, an account with a single latent variable can be rejected in favour of multiple latent variables (Newell et al., 2010). The conjoint monotonic regression test developed by Kalish et al. (2016) can be applied to test for significant departures from a monotonic curve.

As an initial investigation, examples of “disappearing dissociations” were found when STA was applied to two influential ToM studies in Fig. 1; one study compared clinical populations (Shamay-Tsoory et al., 2010), and the other compared different age groups (Bottiroli et al., 2016). Both studies (chosen due to their high citations and the inclusion of necessary descriptive statistics) had identified key dissociations between cognitive and affective ToM. However, Fig. 1 shows that according to STA, the data do not reject that a single latent variable drives performance on the cognitive and affective ToM tasks. These results highlight the need for more rigorous tests of the dimensional nature of ToM.

The current study aimed to use STA to investigate the dimensionality of ToM in a sample of healthy older adults. Previous dissociation evidence has primarily been based on clinical populations or comparing younger versus older adults (Bottiroli et al., 2016; Shamay-Tsoory et al., 2010). However, the current study sought to make pioneering steps in applying STA to more detailed ToM data, examining the dimensionality of ToM for a single group of older adults for which a large dataset was available. As a novel approach, the current study explored whether social exchange type (e.g., sincere or sarcastic exchanges) would have a differential impact on first-order cognitive, second-order cognitive, and affective ToM performance. Social exchange type is a relevant factor for exploring the dimensionality of TOM, based on research demonstrating differential impairments in clinical populations including traumatic brain injury, schizophrenia, and right hemisphere brain lesion patients, with diminished detection of sarcastic but not sincere exchanges (McDonald, 2012). Healthy older adults have also been shown to have impaired detection of sarcastic exchanges, while sincere exchanges were unaffected (Phillips et al., 2015). Additionally, cognitive ToM performance has been found to be more strongly associated with the identification of sarcastic exchanges, as compared with affective ToM performance (Hennion et al., 2015; Li et al., 2017). Given the proposed strong role of emotion perception in affective ToM (Bek et al., 2021; Henry et al., 2006), we also investigated emotion perception as a possible contributing factor to the multidimensionality of ToM. Therefore, we tested whether state-trace analysis (including social exchange type and emotion perception factors) would demonstrate non-monotonic relationships between each of the two orders of cognitive ToM paired against affective ToM and a nonmonotonic relationship between first-order and second-order cognitive ToM.

## Materials and methods

### Participants

The current study examines (in much more detail) part of a larger, 3-hour testing protocol reported in Lavrencic et al. (2016), which examined the association between social cognition and cognitive reserve using summary-only ToM scores. The sample comprised 115 healthy older adults aged 60–85 years (*M* = 68.5, *SD* = 5.92, 61.7% female) recruited from South Australia. One participant was removed from data analysis due to incomplete data, resulting in a final sample of 114. G*Power (Faul et al., 2007) was used to ensure this sample size was sensitive to detect an assumed power of .80, alpha .05, and a partial η^*2*^ of .20 which was reported by Bottiroli et al. (2016) in their analysis of variance investigating ToM dissociations across age groups.

Relevant exclusion criteria for the current study were (1) nonnative English speakers; (2) uncorrected hearing or visual impairments; (3) history of a psychiatric disorder within the past 5 years; (4) any brain disease; or (5) diagnosis of a learning disability (Lavrencic et al., 2016). Participants received an honorarium of $40 AUD each. The study was approved by the University of South Australia’s Human Research Ethics Committee.

### Measures

#### Cognitive and affective ToM

The Awareness of Social Inference Test–Revised (TASIT-R; McDonald et al., 2011) Form A Parts Two and Three, Social Inference Minimal and Social Inference Enriched, were used to measure cognitive and affective ToM. Both Parts required participants to watch a series of short video vignettes of social interactions between trained male and female actors. After viewing each scene, participants answered three probe questions targeting their understanding of what the actors were doing, thinking, and feeling. These “think” questions measured first-order cognitive ToM (e.g., “Does Ruth think Gary should stop what he is doing and help her?”), “do” questions measured second-order cognitive ToM (e.g. “Is Ruth trying to pressure Gary into helping her?”), and “feel” questions measured affective ToM (e.g., “Is Ruth annoyed with Gary?”). Participants answered Yes, No, or Don’t Know for each probe question. Participants also answered a fourth probe question, “say,” a control measure to test their story comprehension; these responses were not examined in the current study. Part Two, Social Inference Minimal, depicted 15 scenes categorized into three different social exchange types: (i) *sincere*, where the target speaker meant what they were saying and all text and paralinguistic cues were consistent; (ii) *simple sarcasm,* where one of the speakers meant the opposite of what they were saying, but this could only be discerned by reading their paralinguistic cues and; (iii) *paradoxical sarcasm* where the dialogue between two speakers only made sense if it was understood that one of the speakers was being sarcastic. Part Three, Social Inference Enriched, depicted 16 scenes categorized into two social exchange types: (i) *sarcastic*, where the dialogue between two speakers only made sense if it was understood that one of the speakers was being sarcastic; and (ii) *lying*, where one of the speakers was lying but intended their message to be accepted as true. In these 16 scenes, additional video footage was portrayed so that extra contextual clues could help inform participants’ answers.

TASIT-R responses were examined as the mean proportion of endorsement (“yes” decisions) for both target and lure items (i.e., participants’ mean response when the correct response was “yes” and “no”, respectively) for each type of probe question (i.e., do, think, and feel) and for each social exchange type (i.e., sincere, simple sarcasm, paradoxical sarcasm, sarcasm, and lying). Note that given TASIT-R uses a yes/no task, to be consistent with a signal detection framework approach, we examined the proportion of endorsements rather than accuracy (i.e., in contrast to the accuracy data in the reanalyses in Fig. 1) and examined responses to target and lure items separately. This approach is consistent with previous STA applications (e.g., Hayes et al., 2018; Stephens et al., 2019). Separating target and lure items also avoids possible averaging artefacts and increases the number of conditions examined, which increases the opportunity to observe departures from monotonicity.^1^

#### Emotion perception

TASIT-R (McDonald et al., 2011) Form A Part One, Emotion Evaluation Test, was used to measure emotion perception. Participants were shown 28 video vignettes of trained male and female actors. Participants were required to choose from seven available emotions, the one they believed was predominantly displayed by the target actor in a scene. These emotions included happy, sad, surprised, neutral, anxious, angry, and revolted. A total score for each correctly identified emotion was first calculated, and then a median split was performed to categorize participants into either high (*M* = 24.92, *SD* = 0.96) or low (*M* = 20.90, *SD* = 2.50) emotion perceptual ability; both groups comprised similar ages (*M* = 67.70, *SD* = 5.09 and *M* = 69.19, *SD* = 6.51, respectively). These scores were fairly consistent with normative data for the comparable age groups (*M =* 23.08, *SD* = 2.42 for those ages 60–74, and *M* = 21.38, *SD* = 2.83 for those ages 75+; McDonald et al., 2011).

### Procedure

Informed consent was first obtained for all participants, and then questionnaire and behavioural data were collected in one-on-one interviews. The order of tests was randomized to avoid fatigue effects; the order of TASIT-R Parts One to Three was held constant. TASIT-R scenarios were played on a computer, and participants responded verbally to all questions.

### Statistical analysis

Statistical analyses were run using R (Version 4.0.2; R Core Team, 2021). The code used for these analyses is publicly available (https://github.com/ALJarvis/STA_ToM). Analyses were based on processing the raw item-level data from individual TASIT-R responses, which were coded as 1 = “Yes” decisions, and 0 = “No” decisions. Out of a possible 93 responses for each participant, 61 responses of “Don’t know” were identified across all participants, accounting for less than 1% of responses (total responses equalled 14,136); these were treated as missing data and removed from further analyses.

To examine whether traditional dissociation tests uncovered interactions for endorsement rates that were suggestive of a multidimensional account of ToM, a preliminary mixed analysis of variance was run using the emmeans package (Lenth, 2022). A 5 (social exchange type) × 3 (think, do, or feel question) × 2 (high or low emotion perception ability) × 2 (correct response of yes or no) design was utilized, with emotion perception examined as a between-subjects factor and all else as within-subjects factors; all used Greenhouse–Geisser corrections to adjust for lack of sphericity. Planned contrasts using Tukey’s corrections were run to investigate any significant interactions.

STA was then run using the STACMR-R package (Dunn & Kalish, 2020) to examine the dimensionality of the TASIT-R data. High versus low emotion perceptual ability, social exchange type, and yes/no correct responses were the experimental factors that defined the conditions (data points) in the state-trace plot. The dependent variables (*x*- and *y*-axes of the state-trace plots) were think, do, or feel responses. The first analysis (“think” versus “feel”) captured first-order cognitive versus affective ToM. The second analysis (“do” versus “feel”) captured second-order cognitive versus affective ToM. The third analysis (“think” versus “do”) captured first-order cognitive versus second-order cognitive ToM. Departures from monotonicity were assessed via conjoint monotonic regression (CMR), which first identifies the best-fitting monotonic approximation of the observed data. Next, a bootstrap procedure is used to test the null hypothesis, which, in this case, is the hypothesis that the monotonic approximation—consistent with a single latent dimension—provides an adequate fit to the data. If *p* < .05 is found, the null hypothesis of a single latent dimension can be rejected in favour of multiple dimensions.

## Results

### Mixed analysis of variance

**Fig. 2:**
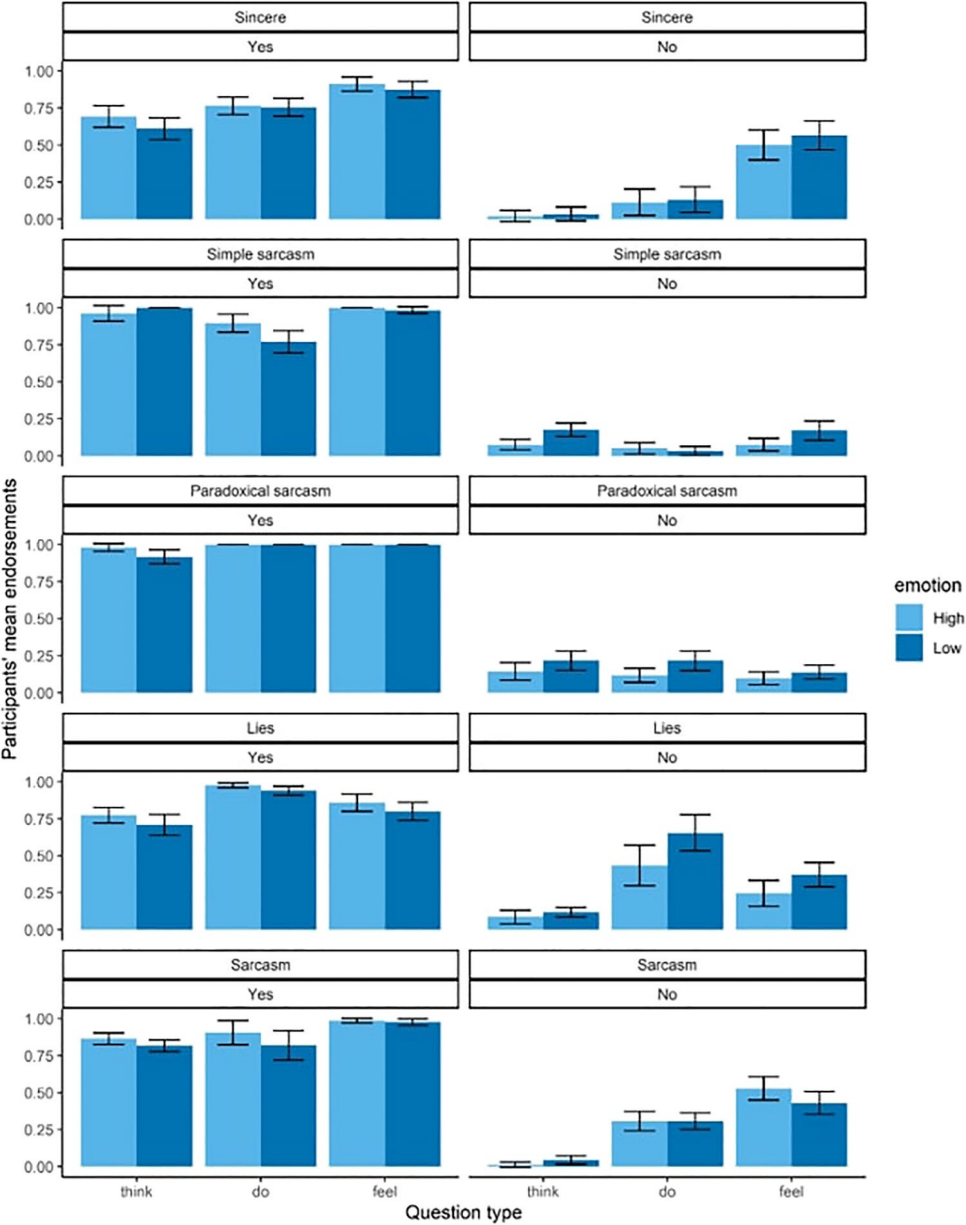
Participants’ mean endorsements for the experimental conditions. *Note.* The experimental conditions are question type (think = first-order cognitive, do = second-order cognitive, feel = affective), social exchange type (sincere, simple sarcasm, paradoxical sarcasm, sarcasm, and lies), emotion perceptual ability (high or low), and item type (correct response of yes or no); error bars represent the standard error of the means

Participants’ mean endorsements on TASIT-R items across each of the experimental conditions are presented in Fig. 2. Results from the mixed analysis of variance revealed that the four-way interaction between question type, emotion perception, correct response (item type), and social exchange type was not significant *F*(5.92, 657.39) = 1.78,* p* = .102, η_p_^2^ *= .*02. There was, however, a significant three-way interaction between question type, emotion perception, and social exchange type *F*(5.78, 641.89) = 3.19,* p* = .005, η_p_^2^ *=* .03, as well as a significant and sizeable three-way interaction between question type, correct response, and social exchange type *F*(5.92, 657.39) = 19.23,* p* <.001, η_p_^2^ *=* .15. Crucially, there were dissociations between think (first-order cognitive), do (second-order cognitive), and feel (affective) responses, consistent with traditional evidence for multiple dimensions. Planned contrasts identified that social exchange type had a relatively limited effect for think-no questions but a large (and differential) effect for do-no and feel-no questions; higher endorsement rates were observed in the lies and sarcasm exchanges for do questions, while higher endorsement rates were observed in sincere and sarcasm exchanges for feel questions. Unlike the “no” item condition, the “yes” condition showed less effect of social exchange type across all three question types, while emotion perception had similarly limited effects.

### State-trace analysis

**Fig. 3:**
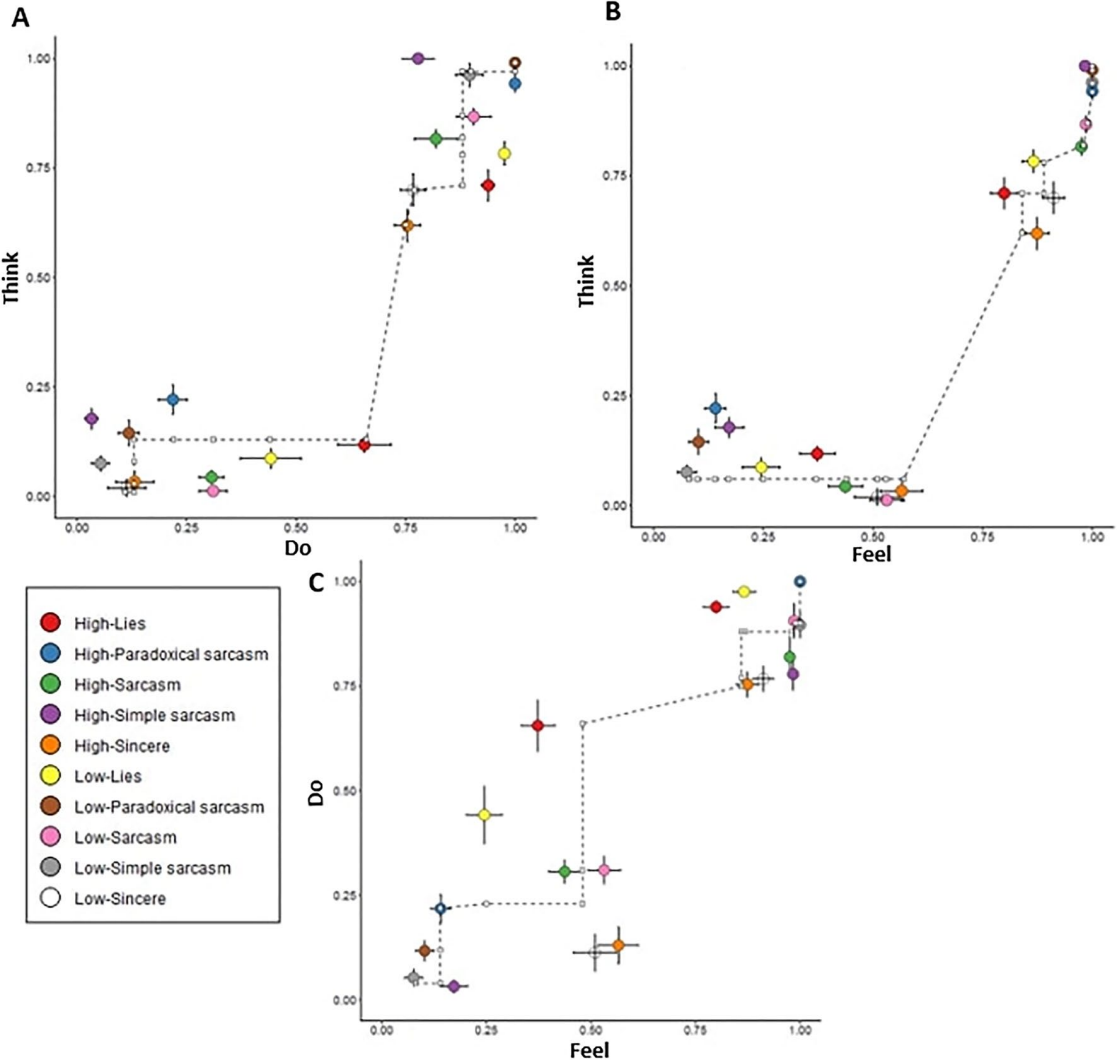
State-trace results. *Note.* The three state-trace plots each depict the mean proportion of endorsements for each social exchange type, correct response item type (“yes” items are clustered in the top right corner; “no” items are clustered nearer the bottom left corner), and emotion perceptual ability category (high or low) for two STA dependent variables examining question type (think, do, or feel). The dashed lines represent the best-fitting monotonic curve, and the error bars indicate the standard error of the mean. (**A**) shows the state trace plot for think (first-order cognitive ToM) by do (second-order cognitive ToM) questions, (**B**) shows the plot for think (first-order cognitive ToM) by feel (affective ToM) TASIT-R questions, and (**C**) shows the plot for think (first-order cognitive ToM) by do (second-order cognitive ToM) TASIT-R questions. All three plots demonstrate nonmonotonic relationships between the two STA-dependent variables, offering evidence against a single-dimensional account of ToM

The three state-trace plots comparing think (first-order cognitive), do (second-order cognitive), and feel (affective) responses are shown in Fig. 3. As seen in all three plots, there is a clear departure from the best-fitting monotonic one-dimensional model, showing evidence for a multidimensional model. This is supported by the CMR model fitting results, where all *p* values were < .001 even after Bonferroni corrections were made. In addition, Fig. 3 shows that the largest departures from monotonicity tended to be for items where the correct response was “no,” with differential endorsement rates across social exchange types, as discussed above.

## Discussion

The three state-trace plots presented in the main study provide rigorous evidence for ToM being a multidimensional construct, as demonstrated through the nonmonotonic pairwise relationships between the three ToM subdomains. These results significantly strengthen previous conclusions about the multidimensionality of cognitive and affective ToM processes from studies using traditional (but problematic) inferential statistics in healthy (Bottiroli et al., 2016; Duval et al., 2011) and clinical groups (Shamay-Tsoory et al., 2010). Furthermore, the demonstrated multidimensional state-trace of first- and second-order cognitive ToM contradicts arguments that the two orders of cognitive ToM represent levels of a single dimension of task complexity (Bibby & McDonald, 2005).

Divergent results were found between the current main study and the reanalyzed STA results displayed in Fig. 1 from Shamay-Tsoory et al. (2010) and Bottiroli et al. (2016). A key reason could be that these two previous studies included only a relatively small number of conditions and independent variables, which limits the opportunity to observe departures from monotonicity (Prince et al., 2012). The ability to detect monotonicity departures was higher in the current study through the examination of multiple experimental conditions based on emotion perception ability, social exchange type, and correct response item type. Additionally, these two previous studies examined ToM using different measures to the current study, while Shamay-Tsoory et al. (2010) also examined individuals with psychopathy. Future research can further explore the generality of our findings across such methodological differences.

The current results show that future ToM research, including ageing and clinical studies, should consider operationalizing ToM using at least these three subdomains: first-order cognitive, second-order cognitive, and affective. Interestingly, some researchers have also distinguished between first- and second-order *affective* ToM inferences, with this distinction measured within the Yoni task (Shamay-Tsoory & Aharon-Peretz, 2007) and Virtual Assessment of Mentalising Ability (Canty et al., 2017). However, a strong theoretical rationale for this distinction is yet to be established. Current definitions for second-order affective ToM appear to combine both cognitive and affective abilities, with the Yoni task describing its measurement of second-order affective ToM to represent the “ability to understand what someone thinks about what someone else feels” (Shamay-Tsoory & Aharon-Peretz, 2007, p. 3059). Such research highlights the need for further investigation of the validity of distinguishing between first- and second-order affective ToM. Nonetheless, recent studies continue to examine ToM either as a unidimensional ability (Benito-Ruiz et al., 2022) or do not distinguish between first- and second-order inferences in their measurement of cognitive or affective ToM (Lin et al., 2021). Therefore, it is hoped the current results will encourage future researchers to explore defining and measuring ToM as consisting of at least first-order cognitive, second-order cognitive, and affective subdomains. Such an approach could help improve both the sensitivity of diagnostic methods and the targeting of potential treatments and interventions for social cognitive impairments.

It is important to note that the statistically significant state-trace analysis results only provide evidence for more than one dimension in the underlying cognitive structure; the specific number and nature of the multiple dimensions cannot be concluded from STA alone (Stephens et al., 2019; also see the discussion by Ashby, 2019; Stephens et al., 2020b). Although the exact nature of the mechanisms that drive each ToM subdomain remains debated, some processes that have been implicated include emotion perception (Bek et al., 2021), executive functioning (Bottiroli et al., 2016), and empathy (Shamay-Tsoory et al., 2010). An important direction for future research will be to investigate whether other processes, including executive functioning and empathy, underlie the different ToM subdomains. While the current study did examine the influence of emotion perception, this was not found to have a strong effect on ToM performance in the current sample. This limited effect could partly be attributable to the level of variability in mean emotion perception scores between the low and high groups or a product of the type of emotion perception measure examined. As noted above, common emotion perception and affective ToM tasks share similar task demands by requiring the identification of emotions or affective states through static images of faces or eye regions (Baron-Cohen et al., 2001; Henry et al., 2015). Given that the current study required participants to identify emotions and affective states through verbal, bodily, and facial cues of trained actors during different social exchanges (McDonald, 2012), the more complex task demands could explain why emotion perception was not found to have a strong relationship with either affective or cognitive ToM task performance. Therefore, the influence of emotion perception on ToM performance should continue to be investigated, especially in clinical samples with more variability in ability level.

A novel approach of the current study was to investigate whether various types of social exchanges were differentially related to cognitive versus affective ToM. Social exchange type was found to have a notable effect on ToM responses, with performance on each social exchange type demonstrating a different response pattern for first-order cognitive, second-order cognitive, and affective ToM, as evidenced in Fig. 2. An avenue of future research will be to continue investigating the role of ToM processes when people assess different social exchange types, which is also important, given that McDonald (2012) found clinical samples to be differentially impaired for different social exchange types.

An important methodological strength of the current study was the use of a single task using the same stimuli to examine cognitive and affective ToM, rather than using separate tasks with different stimuli (e.g., Henry et al., 2015). Measuring both domains using the same stimuli helps ensure that differences in cognitive versus affective ToM performance are being measured, rather than differences in task stimuli or demands. Given the methodological confounds present when examining cognitive and affective ToM using separate tasks, it is suggested that future research also examines both ToM domains using closely matched tasks.

A novel analytic strength of the current study was the application of STA to test for evidence against a single ToM latent variable or dimension. Unlike standard dissociation approaches, which make the strong (and unwarranted) assumption of a linear mapping between latent and dependent variables, the current application of STA makes the milder assumption of only a monotonic mapping (see Loftus, 1978; Stephens et al., 2019; Wagenmakers et al., 2012). If the monotonicity assumption is valid, the multidimensional state traces that we observed are evidence against a single latent variable. However, the monotonicity assumption has been criticized as being invalid for some situations or quantitative models (e.g., Ashby, 2019; Ashby & Bamber, 2022) and is open to review in future research. If the monotonicity assumption is invalid in a given situation, alternative STA tests would need to be developed and applied, or alternative dependent variables may need to be considered (see Dunn & Kalish, 2018). Nevertheless, it is important to note that monotonicity is generally a minimal requirement for a given change in an observed measure to say anything about the underlying theoretical construct (for further discussion, see Dunn & Anderson, 2023; Dunn & Kalish, 2018). For example, without such an assumption, a shift in scores on an emotion perception test could not say anything about the underlying emotion perception ability—it may have increased, decreased, or remained unchanged.

A potential limitation of the current study is that we analyzed group-level performance. As illustrated by Prince et al. (2012), neither monotonic nor nonmonotonic state traces at the individual participant level are necessarily preserved under averaging across participants. The current study included emotion perception ability as a between-subjects factor, but where all factors can be varied within participants, it could also be important to examine individual-level state traces. Note that any limitations with the current averaging approach would be equally true for standard dissociation approaches.

An essential advance in exploring the nature of the underlying processes in ToM will be to specify and test more detailed formal models. As noted previously (e.g., Stephens et al., 2019), a multidimensional state trace result may imply the underlying operation of multiple distinct processes. Alternatively, the result may also be consistent with “single process” accounts that include multiple parameters. One possible multiple-process account is dual-process theory, which proposes two types of cognitive processes or systems; one is fast and intuitive, and the other is slow and reflective (Evans, 2008; Evans & Stanovich, 2013; Stephens et al., 2020a). Such an account is consistent with a study by Bull et al. (2008), which found that cognitive ToM performance was more impaired than affective ToM performance when concurrently completing an executive functioning task, indicating that affective ToM could be more intuitive than cognitive ToM. To further test this idea, quantitative models could be developed based on the signal detection framework, which can instantiate competing dual-process and single-process variants (for an application to inductive and deductive reasoning, see Stephens et al., 2020a). Notably, under this approach, both single- and dual-process model variants can have multiple parameters (e.g., reflecting subjective assessments of the strength of a ToM inference and response thresholds) and can be tested via extensions to STA such as signed difference analysis (Dunn & James, 2003; Stephens et al., 2018).

Lastly, we note that the current study focused on data from older adults with no preexisting neurological or psychiatric disorders. Future work should test the generalizability of the current findings across other populations. It is possible that age may have influenced the results, given that older adults have been shown to exhibit some ToM impairments compared with younger adults (Bottiroli et al., 2016; Sebastian et al., 2012). For this reason, it is worth examining the dimensionality of ToM using STA in healthy younger populations and clinical populations known to exhibit performance deficits.

In summary, the current study provides evidence to support ToM as a multidimensional construct, possibly with multiple processes underlying task performance in at least first- and second-order cognitive and affective subdomains. The use of STA was a novel approach to uncovering the dimensionality of ToM and enabled a more rigorous examination than behavioural research has achieved so far. From a theoretical perspective, our findings will promote greater consistency within the literature on defining and measuring ToM and has important implications for future research into the processes that drive ToM.

### Supplementary Information

Below is the link to the electronic supplementary material.

Supplementary file1 (DOCX 92 KB)

## Data Availability

The dataset for the experiment reported here is available from the corresponding author upon reasonable request.

## Footnotes

1 We confirmed that the current STA results were unchanged when separate STA plots for mean endorsement of yes versus no items were examined; see Supplementary Material.
