# Supplementary material for: Evidence for a multidimensional account of cognitive and affective theory of mind: A state-trace analysis
Source: Mem Cognit. 2023 Nov 28;52(3):525–35. doi: 10.3758/s13421-023-01481-9 (PMC11021350; doi:10.3758/s13421-023-01481-9)

Supplementary Materials

**Supplementary Figure 1:** STA plots each depicting the mean proportion of endorsements for each social exchange type (lies, paradoxical sarcasm, simple sarcasm, sincere, sarcasm) and emotion perceptual ability category (high or low) for no (left hand plots) versus yes (right hand plots) items. The dashed lines represent the best-fitting monotonic curve, and the error bars indicate the standard error of the mean. All six plots demonstrate non-monotonic relationships between the two STA-dependent variables (all p<.05).


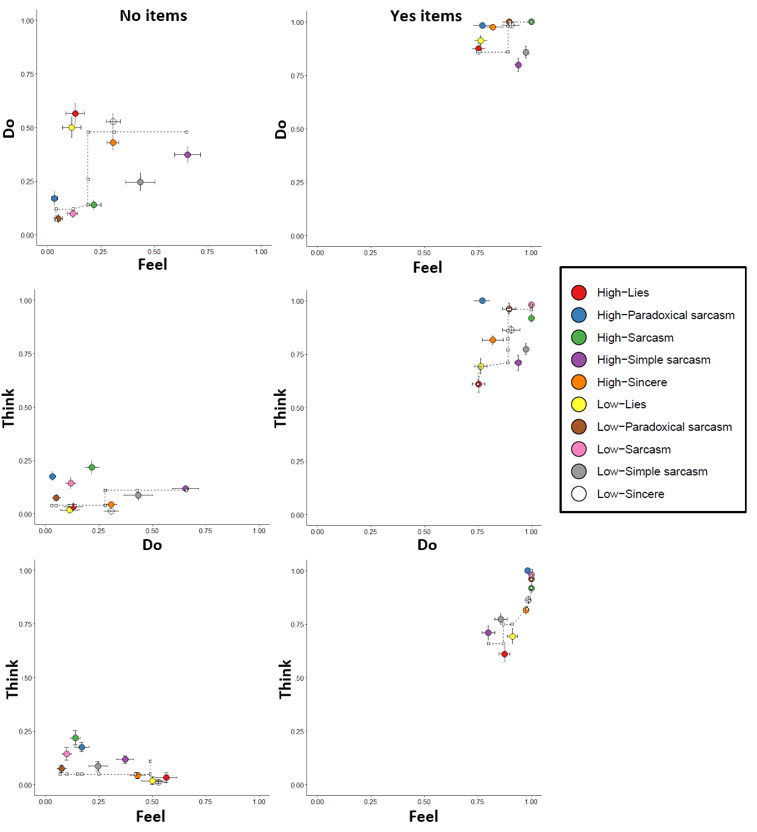

Supplement: Supplementary file 1 — Supplementary file1 (DOCX 92 KB) [file 13421_2023_1481_MOESM1_ESM.docx]
